# Cognitive load, extended reality, and visuospatial abilities in physical science education: a systematic review

**DOI:** 10.3389/fpsyg.2026.1767304

**Published:** 2026-03-13

**Authors:** Conor Desmond Kenneally, Brendan Bentley, John Willison

**Affiliations:** School of Education, Adelaide University, Adelaide, SA, Australia

**Keywords:** cognitive load, extended reality, physical science education, visuospatial abilities, VR/AR education

## Abstract

The application of extended reality (XR) technologies is transforming the education of the physical sciences. These technologies translate elements of a user's lived experience into digital experiences. This study undertook a systematic review using a Preferred Reporting Items for Systematic Reviews and Meta-Analyses (PRISMA) method. The study investigated the relationship between XR spatial presence, user visuospatial processing, and the associated levels of cognitive load (CL). After the initial identification and screening of data, 44 papers were assessed and included in the study. The study found that the application of XR can reduce CL and improve learning outcomes when applied to physical science learning contexts that require the use of visuospatial abilities, where effective implementation is influenced by the level of spatial presence the technology offers. By carefully ensuring congruency between the use of the technology and the goals of the learning task, the results of this review can assist in enhancing task design that utilizes XR.

## 1 Introduction

The adoption of XR technologies represents a potential non-pareil within educational settings to transform the contemporary educational landscape as their adoption becomes increasingly more common (Fegely and Cherner, 2023). As an umbrella term, XR embodies the use of VR, AR, and mixed reality technologies that modify, to varying degrees, the user interface to incorporate, augment, or impose digital elements into it (Bautista et al., 2023). XR technology can be considered as existing along a spectrum with a completely real, physical environment located on one end and a pure VR reality at the other (Çöltekin et al., 2020). VR provides a completely immersive and interactive computer-based simulation of a virtual world. This end of the spectrum essentially embodies a new sense of reality, one that allows the user to access and interact with entirely virtual environments, potentially through all senses available (Alnagrat et al., 2022). Between these two regions of the continuum lies a mixed reality, represented by varying degrees of AR technologies, a term generally used to describe virtual digital objects coexisting in a real environment via their superimposition onto it (Maas and Hughes, 2020). Despite the potential inherent in XR, questions concerning the effectiveness, consistency, and logistics required to implement XR technologies remain largely unanswered (Bautista et al., 2023).

There are substantial inconsistencies in the XR research that addresses the associated learning theories, the inherent effectiveness and the strategies necessary for effective learning, and the teacher training required for successful implementation (McGowan et al., 2023). One exception to this inconsistency is results that show enhanced visuospatial processing by altering a user's perception of reality when applied to learning contexts in the discipline of the physical sciences (Keller et al., 2021; Sun et al., 2019; Wang et al., 2024a). The physical sciences require a heavy demand for visuospatial problem-solving, with ability in this regard correlated with success in these disciplines (Buckley et al., 2018). Visuospatial processing describes the cognitive capacity of an individual to generate and transform spatial information in working memory (WM). As a component of WM, cognitive load theory (CLT) suggests that learning content requiring these skills has the potential to be optimized through careful consideration of the CL implications (Castro-Alonso et al., 2019). Yet broader conclusions, particularly with respect to the degree that XR can immerse the user in the virtual environment and the effect this consequently has on both CL and effective learning, are still relatively unknown (Makransky et al., 2019).

To best understand how the perception of reality can evolve through XR technologies, researchers such as Maas and Hughes (2020) suggest both AR and VR should be considered concurrently. Maas and Hughes (2020) argue that focusing on either VR or AR individually may limit the potential pedagogies that could be established across the manner the technologies consume, create, and manipulate information. To further inform this field of study this paper presents a systematic literature review that critically interrogated the following research questions:

RQ1: Can the application of XR technologies in the learning of physical science content that requires the use of visuospatial abilities reduce CL and benefit learning outcomes?

RQ2: What are the characteristics and properties of successful implementation of XR technologies to alleviate visuospatial CL in physical science education?

Next, the article will provide the background theory of CLT and discuss its application and implications for visuospatial processing when using XR technologies in the physical sciences. The methodology of the review will subsequently be outlined, whereafter the results obtained will be summarized and reported. The article will conclude with an analysis and discussion of data obtained within the context of the proposed research questions.

### 1.1 Cognitive load theory

CLT has been embraced by several domains of research, including psychology and education. It has its origins in instructional theory founded within the parameters of human cognitive architecture (Sweller, 2023). A key element of CLT is the purported impact of the limited capacity that human WM has on acquiring new schema. CLT, informed by human cognitive evolution, can provide the conceptual framing for instructional design that optimizes the efficiency of learning tasks (Richardson et al., 2015). Evolutionary educational psychology models human behavior and cognition through the inherent motivational, cognitive, and behavioral biases humans present when learning (Geary, 2002).

CLT aims to optimize the learning of more complex and abstract knowledge through adopting the major tenets of evolutionary educational psychology and human cognitive architecture. CLT describes three types of load and considers the impact that these impose on the cognitive process of information integration between WM, the structure responsible for processing novel information, and long-term memory (LTM), the principal information store (Castro-Alonso et al., 2024). The first type, intrinsic cognitive load (ICL), is described as the cognitive resources required to understand the inherent complexity of information being processed. The amount of ICL generated is dependent upon the problem-solver's expertise in relation to information already integrated into the cognitive schematic of LTM (Leahy and Sweller, 2008). Extraneous cognitive load (ECL) describes the cognitive resources used to process information that is not related to the complexity of the task itself, most often manifesting through the manner in which new information is presented to the learner (Klepsch and Seufert, 2020). ICL and ECL are imposed by the characteristics of the learning material itself and are additive in their effect (Taylor et al., 2022). WM requires cognitive resources to be available in order to understand the information present in the ICL that is germane to the learning (Sweller, 2010). Germane cognitive load (GCL) therefore represents a productive load that is imposed upon WM as a result of consolidating new information with that which the learner already possesses via schema acquisition (Klepsch et al., 2017).

Insufficient cognitive resources available to dedicate to GCL in schema acquisition, as a result of the additive nature of ICL and ECL, will cause the cognitive system to fail and cease processing information effectively (Sweller et al., 2011). While recent research on GCL has challenged its validity as an independent source of CL, GCL remains a commonly measured construct (Krieglstein et al., 2022). CLT considers how learning tasks can be designed to optimize the allocation of these three different types of CL in WM, as overloading this aspect of cognitive architecture by exceeding its maximum capacity will inhibit the efficacy of a learning task and prevent meaningful learning from taking place (Paas et al., 2010). By designing instructional procedures that reduce WM load, domain-specific schema is able to be more successfully transferred to LTM (Sweller, 2012). A CL effect is therefore said to occur when it is demonstrated that a new procedure empirically produces superior test results, an effect rationalized as a differential in element interactivity between the two procedures (Sweller, 2016).

### 1.2 Visuospatial abilities

WM has been modeled as consisting of a higher-level central executive structure in control of the phonological loop and the visuospatial sketchpad, two lower-level storage components (Castro-Alonso and Atit, 2019). Each of these components is responsible for processing different types of information. The phonological loop processes verbal and auditory information, and the visuospatial sketchpad processes visual and spatial information (Castro-Alonso and Atit, 2019). Through these two separate components, a dual-processing framework has been proposed that involves WM creating and holding both auditory and visual mental representations simultaneously in order to create referential connections between them to draw connections with LTM (Greenberg et al., 2020). When confronted with different, multiple representations of information, these need to be integrated and understood as a coherent whole (Vogt et al., 2021). Coherence and meaning can be thought of as arising both within and between these two systems (Sadoski and Paivio, 2013).

A necessary aspect in adaptation for many higher organisms has been the ability to acquire and retain visuospatial information in memory via the cognitive process of spatial learning (Cassilhas et al., 2016). Responsible for a wide range of cognitive abilities, visuospatial processing refers to the cognitive capacity the human brain has evolved to generate and transform visual and spatial information in WM (Castro-Alonso, 2019). These visuospatial skills are commonly organized into three main categories: spatial perception, spatial visualization, and mental rotation (Trojano et al., 2018). As each requires the processing of visual and spatial information, they will all be associated with, and dependent upon, the visuospatial sketchpad component of WM (Castro-Alonso and Atit, 2019). As a component of WM, these are significantly limited in terms of capacity, yet remain separate from the capacity of the auditory component (Greenberg et al., 2020). Considering CLT as a framework in this context therefore suggests that there are two potential mechanisms to circumvent these limitations to visuospatial processing in WM: unnecessary visuospatial processing could be reduced, or the total visuospatial processing capacity could be increased (Castro-Alonso et al., 2019).

### 1.3 Differentiation of XR technologies

The advent of technological development in recent years has increased the prevalence of XR technologies in both its application to learning and training, as well as in educational research itself (Xi et al., 2023). With the ability of technologies along the XR spectrum to radically and cost-effectively transform the logistics of what is possible in a classroom environment, inferences have been drawn regarding its potential as a paradigm-shifting technology, comparable to the emergence of smartphones, the internet, and tablet computers in terms of impact (Fegely and Cherner, 2023). Yet the terminology used along this spectrum remains contested. Much of the terminology has not been standardized, with many variations existing in its interpretation throughout the literature. Clear differentiation between an AR and a VR learning intervention is therefore required in order to compare and contrast their application. By doing this, the suggestion that XR-based learning has the potential to offer powerful learning experiences through the differing levels of immersion, visualization, and ability to transform a user's sense of space can be interrogated (Çöltekin et al., 2020).

AR tends to be categorized through its ability to impose virtual computer-generated images onto a real environment. Whilst commonly implemented using handheld devices, more immersive modes can be utilized (e.g., by using headsets), yet visual elements of the real world remaining accessible to the user is a defining feature (Mazzuco et al., 2022). Peeters et al. (2023) provide even more stringent parameters for defining AR beyond only overlaying virtual and real objects, further necessitating the need for real-time interactions and requiring the utilization of three-dimensional objects. Comparatively, VR's defining feature is the removal of essentially all real-world visual objects in the pursuit of immersing the user into an entirely virtual one. This increased level of immersion quite severely restricts how VR can actually be implemented, almost exclusively requiring the use of head-mounted displays. Yet the mode of implementation itself is insufficient alone to differentiate between the two, as overlap exists, necessitating deeper consideration.

In the context of XR technologies, current research has begun to investigate how the illusion of immersion and presence interfaces with human cognition via one's perception and sensory systems, with the suggestion being that both motor systems and perceptual systems will influence the construction of concepts (Radianti et al., 2020). VR's ability to generate self-experience in individuals through this sensation of complete immersion has a powerful potential to create a potent sense of presence in the virtual environment it creates (Armougum et al., 2019). Presence in this context refers to the subjective feeling the user experiences from the sense of spatial immersion as a measure of their emotional involvement (Huang et al., 2020), a factor that can be measured both psychologically and physiologically (Marucci et al., 2021). This powerful role of presence through the complete removal of the visual physical world can therefore ultimately be used as the framework through which VR and AR technologies can be differentiated.

### 1.4 Cognitive load implications for XR technology

In its application, AR has demonstrated its effectiveness in optimizing visuospatial processing by supporting the spatial organization processes that need to be undertaken by the visuospatial sketchpad (Costello and Tang, 2007). This is particularly important for the type of visuospatial problem-solving required in many aspects of studying the physical sciences, e.g., in the study of astronomy as an area of physics education, the application of AR as a learning tool has demonstrated its benefit in improving learning (Altmeyer et al., 2020). Yet other studies in the same learning area utilizing the same category of AR technology observed it had no impact (Seo and So, 2022), or even a negative one (Thees et al., 2022). Research into VR technology has similarly begun to reveal its potential benefit in the application to physical science education, e.g., in a chemistry application requiring geometric problem-solving, albeit inconsistently (Laricheva and Ilikchyan, 2023; Won et al., 2019). Much of the discourse around VR's current effectiveness in implementation focuses on the amount of information that the technology can impose upon a learner, with suggestions being made about the potential for cognitive overload (Albus and Seufert, 2022).

The abstractness of physical science knowledge and the invisibility of underlying processes make the effort of learning this form of knowledge even greater than in less-abstract fields, and the difficulty is mirrored in teaching it. It is hypothesized that successful XR use will be able to facilitate the optimisation of CL imposed by tasks with demanding visuospatial processing requirements, with broader conclusions for successful implementation, e.g., with respect to the influence of the specific task type (Kisker et al., 2025), aiming to emerge from this systematic review of the literature. Optimisation of CL can be considered through XR's ability to reduce element interactivity and therefore the cognitive load demands on WM (Wenk et al., 2023). One hope for XR is that it provides a greater “visibility” to invisible processes (Salmi et al., 2017), reducing the requirements of learners needing to imagine these processes (Ganz and Lecon, 2020) to potentially facilitate CL reduction during the learning experience. Yet the benefit of illuminating the invisible with XR remains contested, with assertions instead being drawn not on its ability to develop attainment but instead in promoting engagement and critical thinking skills (Barrow, 2022). The benefit of XR has also been considered and investigated through its potential to offload CL via embodied cognition and the sensorimotor experiences inherent when utilizing the technology (Suo et al., 2025). The assertion of whether the effectiveness of learning principles originally developed for much less immersive multimedia environments can even be generalized to XR technologies like VR, with high levels of presence and immersion, has also arisen (Makransky et al., 2019). Despite its emergence, the use and research into XR and the consideration of its impact through the framework of CL in multimedia learning remain minimal (Bautista et al., 2023), necessitating the need to investigate more deeply and holistically. To further inform this area of research, this study undertakes a systematic review to investigate the relationship between CL, visuospatial abilities, and the application of XR technology in physical science education.

## 2 Method

This systematic review was implemented according to the 2020 PRISMA (Preferred Reporting Items for Systematic Reviews and Meta-Analyses) criteria in the aim of achieving a comprehensive review of the relevant body of evidence (Page et al., 2021). No funding was received to carry out this research.

### 2.1 Eligibility criteria

To ascertain the eligibility criteria of this systematic review, the PICO framework was adopted to methodically dissect the research questions and ensure validity throughout both the conceptual framework and the methodology selected (Møgelvang and Nyléhn, 2023). The inclusion and exclusion criteria were determined based on consideration of the studies' population, intervention, comparator, and outcome (refer to Tables 1, 2). In addition to these parameters, the studies also needed to be published as papers in peer-reviewed academic journals, published in English, and available to the researchers.

**Table 1:** Review inclusion criteria.

**Inclusion criteria**	**Description**
Inclusion criterion 1	The paper is returned by the search terms in Table 3.
Inclusion criterion 2	The paper utilizes an experimental method comparing variables that implement one or more different XR interventions.
Inclusion criterion 3	The paper provides a learning experience in the discipline of the physical sciences.
Inclusion criterion 4	The results of the paper can be analyzed through the frameworks of CLT and visuospatial abilities.

**Table 2:** Review exclusion criteria.

**Exclusion criteria**	**Description**
Exclusion criterion 1	The paper was not published in a peer-reviewed academic journal or was one of the following: conference, symposium or workshop proceedings, dissertation or thesis, review or meta-analysis, or book chapter.
Exclusion criterion 2	The paper was not written in English.
Exclusion criterion 3	The paper was unavailable.

*Population:* The paper was required to provide a learning experience in the discipline of the physical sciences. Any gender or age was deemed appropriate for inclusion as long as the learning intervention embodied a topic in the physical sciences.

*Intervention*: The paper was required to provide an experience with an XR technology (as defined in section 1.3 of the literature review) intervention in some form within the context of a learning experience in the discipline of the physical sciences.

*Comparator*: Comparison of the intervention was required in contrast to a control group. This could manifest as a comparison of an XR intervention with a non-XR intervention, or the comparison between two different implementations of XR technologies.

*Outcome:* The results of the paper were able to be analyzed through the framework of CLT and allowed implications to be drawn with respect to the impact of the technology on visuospatial abilities in order to draw conclusions regarding its effectiveness in improving student outcomes.

### 2.2 Data sources, search, and selection strategy

Literature searches were carried out to identify papers using the Web of Science (WoS) (Clarivate, London, UK), EBSCOhost (EBSCO Industries Inc., Birmingham, AL, USA), Scopus (Elsevier, Amsterdam, Netherlands), ERIC (Institute of Education Sciences, Washington, DC, USA), and PsycINFO (American Psychological Association, Washington, DC, USA) databases, chosen as recognized and relevant sources in the field of educational research (Buchner et al., 2022). Search results were initially refined using keywords related to the main frameworks of this review: CLT, XR technology, and visuospatial abilities in physical science education. Initial conditions of the search needed to incorporate the scope of different types of XR technologies that consideration of the lack of standardized vocabulary. Similarly, CLT needed to be considered to encapsulate research into all the different types, i.e., total CL as well as measures of individual ICL, ECL, and GCL. A similar approach was undertaken to ensure a consideration of visuospatial abilities had been included. However, initial investigation revealed challenges in this regard due to the various terms, phrases, and spellings that represent this body of research, including “visuospatial,” “visual-spatial,” “visual,” “spatial,” “visualization,” “visualization,” “mental rotation,” “spatial perception,” “spatial visualization,” and “spatial visualization.” Finally, application within physical science education was a requirement. Refinement of these factors allowed for the selection of the keywords required to holistically embody each factor (refer to Table 3), with the final search term defined as follows: (“virtual” OR “augmented” OR “extended” OR “mixed”) AND “reality” AND (“cognitive” OR “intrinsic” OR “extraneous” OR “germane”) AND (“visu^*^” OR “spatial” OR “rotation”) AND (“chem^*^” OR “phys^*^”* OR “STEM” OR “science” OR “physical sciences”)*. Parameters were also set regarding the exclusion criteria (refer to Table 2). These included the need for papers to be published in English and available to the researcher at the time of the last database search that was undertaken on the 18 July 2025.

**Table 3:** Keywords used to embody each framework of the systematic review.

**Factor**	**Search terms**
XR Technology	(“virtual” OR “augmented” OR “extended” OR “mixed) AND “reality”
	AND
Cognitive Load Theory	“cognitive” OR “intrinsic” OR “extraneous” OR “germane”
	AND
Visuospatial Abilities	“visu^*^” OR “spatial” OR “rotation”
	AND
Physical Sciences	“chem^*^” OR “phys^*^” OR “STEM” OR “science” OR “physical sciences”

### 2.3 Data type, inclusion, and extraction

Harvested data consisted of

a. *Identification*: year of publication, journal published in, journal impact factor.
b. *Methods*: methodological design, characteristics of implementation, forms of data collection.
c. *Population*: age, academic context.
d. *Interventions*: form of XR technology utilized, comparator used by control group, physical science learning context.
e. *Outcomes*: statistically significant results and conclusions for participant learning, statistically significant results and conclusions for the level of CL experienced by the participant, implications regarding the impact of the technology on participant visuospatial processing.

**Figure 1:**
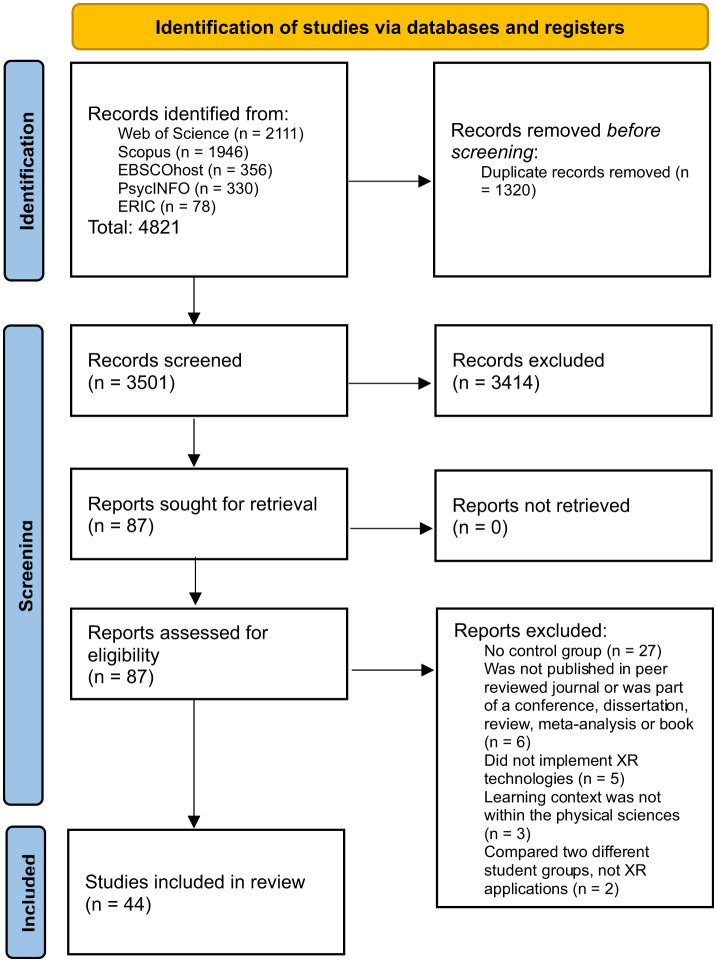
Flow diagram record of included searches according to PRISMA 2020 convention.

Of the 4,821 studies initially located, 1,320 were removed as duplicates. The remaining 3,501 studies underwent analysis of each paper's title, abstract, and keywords with respect to the inclusion and exclusion criteria previously specified (refer to Tables 1, 2). A total of 87 papers were selected for analysis of the whole text, undertaken by two researchers using Covidence software to reach consensus on inclusion or rejection. Forty-four studies were ultimately included for data extraction in the systematic review (refer to Figure 1).

Studies that reported statistical significance of measures of student learning for XR interventions when compared to a control were recorded as having a “positive,” “negative,” or “non-significant” effect. This initial classification allowed for a generalized benefit to be inferred allowing each article's parameters to be specified for learning outcomes. These parameters were used in subsequent analysis. Analysis and summarization of data, and descriptive analysis based on each study's learning outcomes, allowed for a nuanced investigation into the conclusions drawn regarding the benefits XR can yield.

CL measurements were implemented in one of two ways in the included studies; either as measurements of total CL or individually as ICL, ECL, and/or GCL (or combinations thereof). A “positive effect” was recorded if a reduction in total CL, ICL, or ECL was reported, or an increase in GCL. If the inverse was the case, then a “negative effect” was reported (Sweller, 2010). Note that the reporting of quantitative data was not a requirement for the studies' inclusion; rather, an interpretation of results through a CLT framework was, where a number of studies (*n* = 26), with respect to their conclusions on CL (as opposed to learning outcomes), were included as theory-informed interpretations only in this respect.

### 2.4 Bias

**Figure 2:**
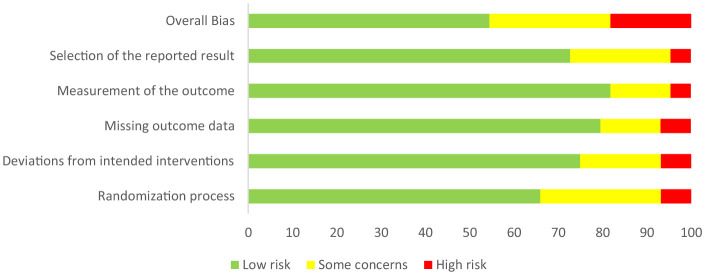
Risk of bias visualization with parameters reported as percentage of studies fulfilling risk criteria.

The potential for bias was mitigated by implementing this systematic review according to the 2020 PRISMA (Preferred Reporting Items for Systematic Reviews and Meta-Analyses) and by strictly following the inclusion and exclusion criteria and screening processes. Any discrepancies in the full-text screening between the two reviewers were resolved through discussion. Studies were evenly weighted, with a risk of bias assessment conducted using the Cochrane Risk of Bias 2 (RoB 2) (Cochran, London, UK) tool to identify limitations and increase the reliability of the assessment undertaken (Babić et al., 2024). The risk of bias assessment revealed trends for overall bias and across five domains. These trends are indicated in Figure 2. The RoB 2 tool (Sterne et al., 2019) indicated a low risk for the overall bias category and all other five domains (refer to Figure 2).

Consideration was given to the statistical significance of the results attained in each paper, regardless of the probability level of significance that was predetermined and of the sample size investigated. Effect size was not reported on for the purposes of this review due to inconsistencies in its reporting throughout the included studies. Quality ratings of methodology, results, and analysis were not employed, meaning that studies with more trustworthy outcomes were weighted the same in this review as those that may have been less trustworthy. The variability of contexts represented in the studies (primary to post-tertiary) and the relatively small number of studies available in this emerging area of research informed the above decisions to not exclude or weight differently. Together, the above means that there was a risk of including false positives in, and excluding false negatives from, the results.

## 3 Results

**Figure 3:**
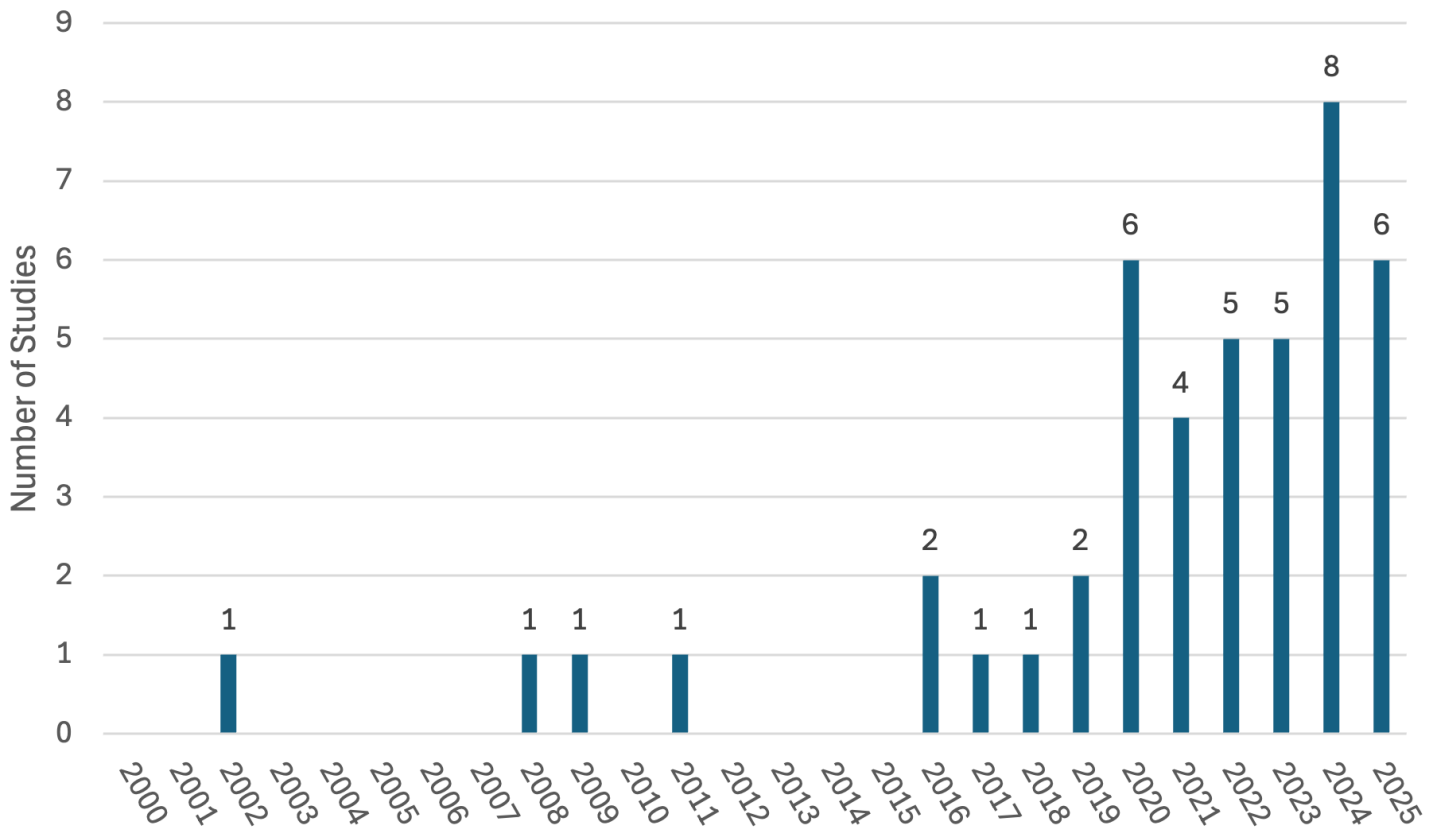
Frequency of studies published by year.

Growth in the number of publications research within the publication date parameters of the analyzed studies (refer to Figure 3) indicates 77% (*n* = 34) of research papers were published after January 1, 2020. Of all the studies analyzed, 65% were implemented at a tertiary level (*n* = 29 with university-level participants), 25% at a secondary level (*n* = 11 with participants in the 7th grade of high school or above), 7% at a primary level (*n* = 3 with participants below the 7th grade), and 2% at a post-tertiary level (*n* = 1 with participants who had graduated university). At the time of publication, 66% (*n* = 29) of the articles were published in a Q1 ranked journal [as per SCImago Journal Rank (SJR)].

**Figure 4:**
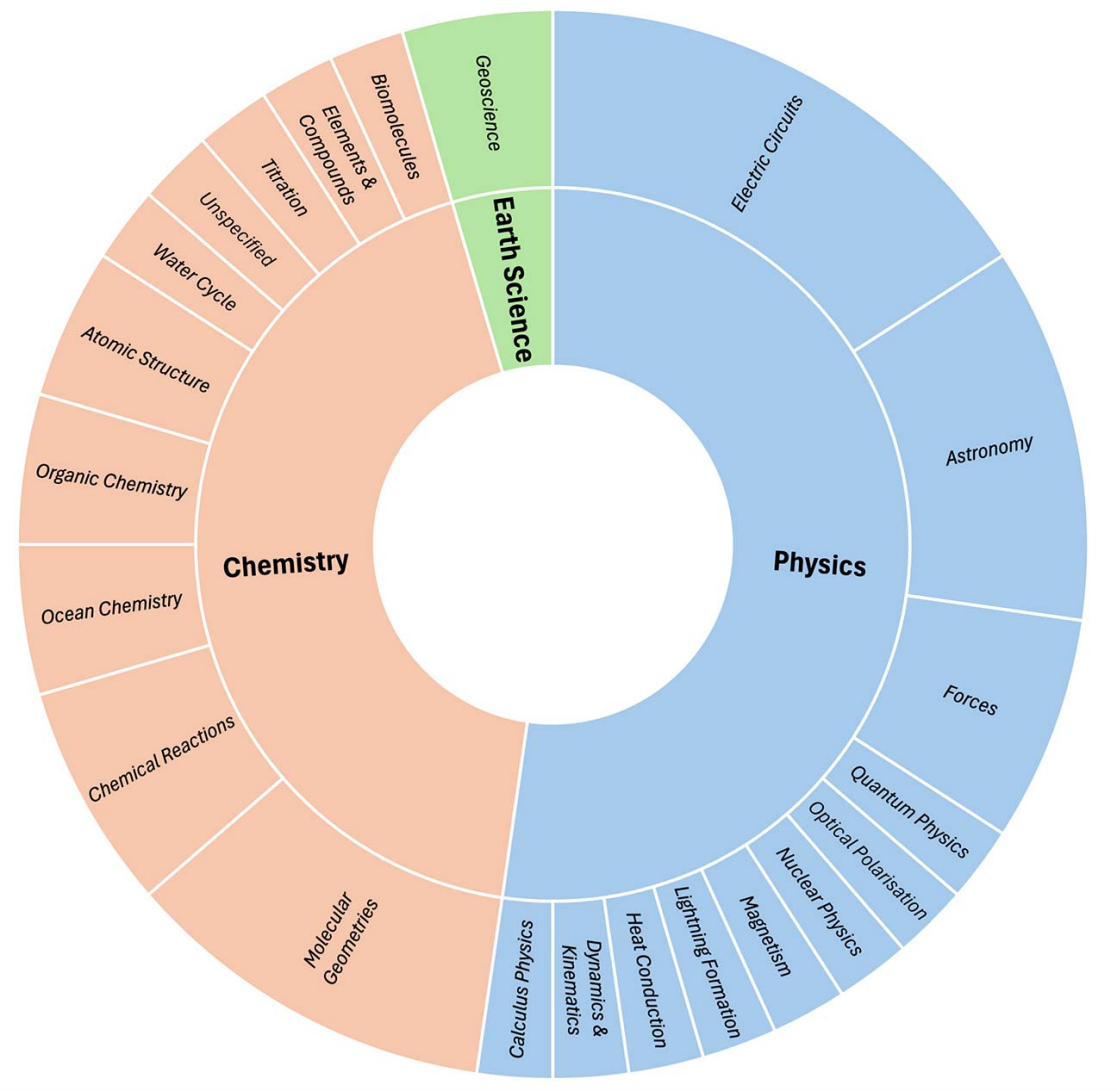
Summary of physical science discipline and subsequent learning topic focus.

Physics was observed to be the most frequently implemented discipline (*n* = 23 with 61% of these implemented at the tertiary level), followed closely by Chemistry (*n* = 19 with 68% of these at the tertiary level). Only *n* = 2 studies were observed to be implemented for the Earth Sciences; both being undertaken at the tertiary level (refer to Figure 4).

**Figure 5:**
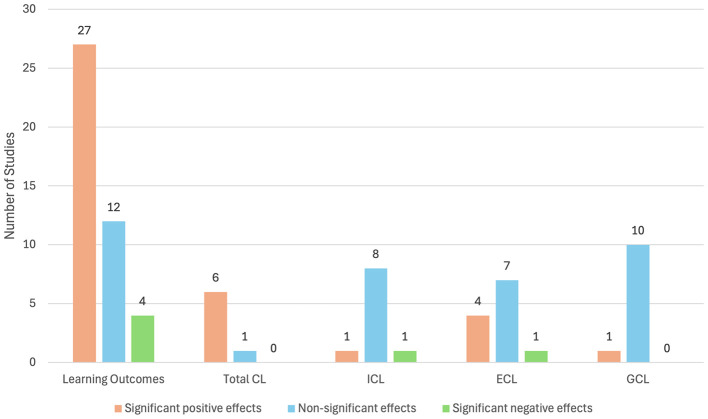
Results of statistically significant outcomes reported.

The systematic review interrogated the statistically significant results reported when an XR learning intervention was compared to a control (refer to Figure 5). Note that the use of “positive effects” in the reporting of CL results refers to a benefit observed in learning, in contrast to “negative effects” referring to a detrimental learning experience.

The conclusions reported by each study were analyzed to ascertain the impact that XR technologies can have on learning outcomes and CL. From the data set, 43% (*n* = 19) were categorized as comparing an AR-supported learning condition to a non-AR condition (refer to Table 4), 27% (*n* = 12) were categorized as comparing a VR-supported learning condition to a non-VR condition (refer to Table 5), and 30% (*n* = 13) were categorized as comparing between two different XR-supported learning conditions (refer to Table 6). Note that the tables specify if the specific variable was not directly measured. In these cases, the studies are included as theory-informed interpretations as per the systematic review's inclusion criteria.

**Table 4:** Extracted data items for studies that compared an AR-supported learning condition to a non-AR condition.

**Study and discipline**	**Significant effects of AR technology**	**Conclusions on learning outcomes**	**Conclusions on CL**
- Altmeyer et al. (2020) - Physics (electric circuits)	- Positive impact on learning outcomes. - No impact on ICL, ECL, and GCL.	- Learning gains detectable for the concept of parallel circuits. - Effective AR-supported learning does not necessarily require specific equipment (e.g., smart glasses); tablet devices can be used to foster conceptual knowledge.	- Temporal contiguity fulfilled in both experimental groups; thus, concluded it is probable the split-attention effect was so small that it was not noticeable in the AR group. - Both groups rated ECL very low, suggesting a floor effect covering possible group differences. - Suggest that instead, the increase in learning outcome could be a result of AR triggering more generative processing.
- Arymbekov et al. (2024) - Physics (nuclear physics)	- Positive impact on learning outcomes. - CL not directly measured.	- Examining pre-test sensitivity revealed that undertaking a pre-test did not have an effect on students' academic success. - AR group observed to have improved motivation for learning physics (also not affected by pre-testing) and fostered a positive attitude toward the subject. - AR provided an immersive experience contributing to a deeper understanding of theoretical concepts.	- The interactive nature of AR could help visualize complex physics phenomena, potentially alleviating visuospatial working memory load to make concepts more tangible and accessible. - The novel element of the tech adding excitement to the learning process was able to capture students' attention.
- Elford et al. (2023) - Chemistry (chemical reactions)	- No impact on learning outcomes. - No impact on ICL, ECL, and GCL.	- No differences observed between experimental groups, yet intra-group improvement observed for the AR group still implying benefit to learning.	- Higher prior conceptual knowledge reported higher measures of ECL, potentially implying that as learner expertise increases, it may be beneficial to place a heavier emphasis on problem-solving.
- Elford et al. (2022) - Chemistry (molecular geometries)	- No impact on learning outcomes. - No impact on ICL, ECL, and GCL.	- No differences observed between experimental groups, yet significant gender differences observed where males performed better. - Qualitative analysis revealed perceived benefits in improved feelings of engagement and convenience compared to utilizing the physical molecular models.	- ICL should not be influenced by learning support such as the use of AR technology. - Gamification elements of the learning task may have required significant cognitive resources that offset the benefits to ECL that AR may imbue. - Non-significant difference in GCL of groups suggests it is indicative of information retention.
- Habig (2020) - Chemistry (molecular geometries)	- Positive impact on learning outcomes. - CL not directly measured.	- Benefit of AR technology in solving items revealed sex differences that were confirmed for male students only. - In contrast, females scored higher on the 2D figures utilized in the non-AR group.	- The combining of textual and visual information during the learning task posed to be a cognitively demanding process that imposes high cognitive load expenses. - This would be exacerbated even more so by students with low spatial abilities.
- Keller et al. (2021) - Chemistry (organic chemistry)	- No impact on learning outcomes. - Positive impact on ICL and ECL. - No impact on GCL.	- Prior knowledge was a predictor of success in the non-AR group but not the AR group. Suggests the potential for AR to reduce the influence of prior knowledge on learning success. - Assume that the impact of AR-based learning may increase with the complexity of the learning topic.	- AR has the potential to reduce the influence of mental rotation abilities on CL, especially if topics are being introduced to students for the first time. - The better the students rated the app, the lower level of ECL observed. Implies the negative impact that a poorly designed system could have in contributing to overload.
- Kenneally and Bentley (2024) - Chemistry (molecular geometries)	- Positive impact on learning outcomes. - Positive impact on total CL.	- The non-AR group were unable to engage in mental rotations as WM resources depleted during the instructional phase when attempting to rotate mentally, rather than transferring new schemas. - In contrast, the AR group were able to apply mental rotation visualizations more freely.	- Processing and drawing on referential connections between multiple representations imposes high cognitive effects. - The AR group yielded lower CL expenses that encouraged them to construct schemas optimizing the relationship between mental rotation and achievement.
- Laumann et al. (2024) - Physics (optical polarization)	- No impact on learning outcomes. - Negative impact on ICL. - No impact on total CL, ECL, and GCL.	- Suggest the non-AR environmental design compensated for potential drawbacks, thus reiterating the importance of good instructional design independent of technological implementation. - AR group did express higher levels of interest, enjoyment, and motivation to learn; however, one cannot exclude the impact of novelty effects.	- The difference in ICL suggested to be due to differences in prior knowledge. - It was also considered that the non-AR group did not need to reflect on the additional visualizations imposed in the AR environment, potentially reducing the cognitive expenses required to complete the task.
- Ling et al. (2021) - Chemistry (organic chemistry)	- No impact on learning outcomes. - CL not directly measured.	- The outcomes of learning via AR will vary from person to person, and so there needs to be careful consideration of the different types of learners in the design of a learning task. - The conditions required for good lasting learning outcomes are more demanding than for good immediate learning outcomes.	- Students without a solid learning foundation but with high spatial abilities can benefit from AR technology to reduce ECL. - If they also do not have high spatial abilities, it was concluded that AR could improve GCL (with reservations).
- Liou et al. (2017) - Physics (astronomy)	- Positive impact on learning outcomes. - CL not directly measured.	- Outperformance of AR linked to perception of immediacy in students' sense of a realistic context, promoting an increase in concentration. - Lack of connection in the non-AR group yielded lower-quality interactions with the real environment, causing a lower sense of immediacy and concentration. - The AR group also outperformed in measures of usefulness and attitude.	- The AR system integrated with the real environment allowed easier referential connections with the real world. This being shown simultaneously on screen caused a decrease in the mental load provoked. - In contrast, the non-AR group had no connection of instructional material with the environment, yielding lower-quality interactions with the real environment.
- Liu et al. (2021) - Physics (magnetism)	- Positive impact on learning outcomes. - Positive impact on total CL.	- Students in the AR condition were more able to process the complexity of the experimental task. - The use of a real object-based marker (i.e., instead of an image marker) can incorporate their own characteristics into the experience and provide multichannel interactions to positive effects.	- The AR tool imposed the lowest experimental information interaction on students for both mental load and mental effort. - The use of AR facilitated a reduction in CL by allowing abstract concepts to be transformed into concrete ones by providing natural interactions.
- Olea-Ibarra et al. (2025) - Physics (astronomy)	- Negative impact on learning outcomes. - CL not directly measured.	- In this case, AR appears to have inhibited learning, despite more positive emotions observed in the AR group. - It is suggested that immersive media may be detrimental if they introduce emotions that detract from the task, in contrast to the less stimulating non-AR tech that may allow focus to be kept on the task.	- More emotional arousal could overload learners and inhibit learning. - Possible overload of learners; therefore, may be caused by the emotionalising effect of the medium more so than how the information was presented.
- Singh and Kaur (2025) - Chemistry (molecular geometries)	- Learning outcomes not directly measured. - Positive impact on total CL.	- The AR group suggested performing better than the non-AR group through the 3D visualizations allowing the possibility to interact and experience the virtual content.	- It was concluded that AR technology had positively influenced student CL, where students in this group exhibited a lower level of cognitive resources to understand the specific chemistry terms used in the course.
- Sotiriou and Bogner (2008) - Physics (Forces)	- Positive impact on learning outcomes. - CL not directly measured.	- Visualizations from the AR technology rationalized to help students correct common misconceptions by adding dimensions of importance to the exhibit and helping them recognize interconnections between issues discussed and relevance to their lives.	- No difference was observed in the measurement of effort between the two groups, implying that the AR group did not exhibit any greater level of CL and was implemented as a user-friendly tool. - AR may allow students to not only rely on the information provided to them but also on that provided by themselves.
		- AR can provide stronger context and develop interest due to its opportunity for precise measurements, deeper personal experience, and visualization of usually unseen factors.	
- Thees et al. (2022) - Physics (electric circuits)	- Negative impact on learning outcomes. - No impact on ICL, ECL, and GCL.	- It was suggested that the limited view of the head-mounted display in the AR group impeded learning, as participants had to actively engage in turning their view to obtain relevant data. - Discuss the choice of AR device in the effectiveness of the technology but more actively conclude that there may be a mismatch of the affordances of the chosen device and the cognitive processes that were intended to be triggered to enable successful learning. - There is a need to decide if global coherence or local coherence formation is required, with AR potentially being more appropriate in the former.	- It rationalizes the formation of global coherences requiring the integration of separate local coherences into a coherent single representation, something that imposes very high CL expenses, but which yields deep knowledge structures. - In this AR environment, they suggest that spatial contiguity failure occurred due to poor spatial proximity of information, making it difficult for students to form initial local coherences. - In contrast, the non-AR version allowed simplified local coherences to be formed without performing resource-consuming search processes.
- Thees et al. (2020) - Physics (heat conduction)	- No impact on learning outcomes. - Positive impact on ECL. - No impact on ICL and GCL.	- It is suggested the interplay between the scientific theoretical background relevant to the experiment and the interpretation of the observations during the analyses is crucial for conceptual knowledge acquisition in this context. - Students may only acquire new knowledge structures when utilizing AR technology after in-depth analyses of the measurement data.	- The appearance of information as multiple external representations did not lead to a higher level of complexity of the content but was successful in reducing the level of ECL exhibited by the AR group. - Multiple external representations and temporal contiguity principles seem to be linked, where simultaneous presentation of corresponding sources of information allowed learners to recognize and process their interdependencies.
- Utami et al. (2020) - Chemistry (elements and compounds)	- Positive impact on learning outcomes. - CL was not directly measured.	- AR problem-based learning was concluded to increase students' metacognitive skills to improve learning and problem-solving ability. - It is suggested AR is able to evoke higher-order creative and critical thinking skills, as well as evoke student motivation, to the benefit of learning.	- The use of AR technology may support the process of searching for information in order to build explanations required to solve the problem correctly.
- Wan et al. (2018) - Chemistry (atomic structure)	- Positive impact on learning outcomes. - CL not directly measured.	- Use of AR was successful in improving learning outcomes, suggesting that all learning types (visual, auditory, kinesthetic) should be implemented into instructional materials to leverage the learning modalities to improve the effectiveness of learning. - Participation in the use of AR tech was high, with students being excited and having fun, suggesting its ability to improve attitudes toward learning.	- Did not only focus on the visual benefits of AR but also the auditory and, to a lesser extent, kinesthetic supports that it may offer in cognition.
- Zatarain-Cabada et al. (2023) - Physics (dynamics and kinematics)	- Positive impact on learning outcomes. - CL not directly measured.	- The AR tech utilized was rationalized to have met the four factors measuring students' perceptions affecting their motivation; it is attractive for learning, capable of appealing to the interest and enthusiasm of students, meets students' expectations, and satisfies their needs and curiosity for learning, from an organized, interactive, and easy-to-use didactic medium.	- The novelty effect of the technology, whilst having a positive impact on attention as a factor, had a negative effect on students' confidence by overwhelming them during first impressions using the technology.

**Table 5:** Extracted data items for studies that compared a VR-supported learning condition to a non-VR condition.

**Study and discipline**	**Significant effects of VR technology**	**Conclusions on learning outcomes**	**Conclusions on CL**
- Amirbekova et al. (2024) - Chemistry (unspecified)	- No impact on learning outcomes. - CL not directly measured.	- Cognitive ability criteria, concentration, and abstract thinking, promoted in the VR group but observed without a corresponding increase in academic performance. - Recommend VR tasks to be developed to require the application of cognitive skills to problem-solving and that impact may be more pronounced long-term. - VR group also experienced lower levels of depression and anxiety.	- Immersive learning may improve cognitive skills, but academic success is still influenced by other factors such as assessment effectiveness and task complexity.
- Crogman et al. (2025) - Physics (calculus based physics)	- Positive impact on learning outcomes. - CL not directly measured.	- VR may enhance engagement associated with understanding physics concepts without disproportionately benefiting any subsets of students. - Technology can fulfill the Kolb cycle; provides stimuli to enhance the depth of learning, offers realistic simulations to engage and reflect with, abstract conceptualization is enabled to construct mental models that can then be applied to fulfill active experimentation.	- VR may be able to support the learning process to foster deeper and more uniform understandings of the material through multisensory learning.
- Ernawati and Ikhsan (2021) - Chemistry (titration)	- Positive impact on learning outcomes. - CL not directly measured.	- Use of the VR technology improved cognitive achievement throughout the chemistry experimental procedure undertaken. - Similarly saw benefits in additional measures such as scientific attitude and enthusiasm.	- The utilization of VR technology supported the cognitive domains outlined in Bloom's taxonomy; it allowed visualization, understanding of the materials used, application of the experimental procedure, analysis of data, evaluation of conclusions, and creation of supporting materials.
- Keles et al. (2023) - Physics (forces)	- Positive impact on learning outcomes. - CL not directly measured.	- It was concluded that the use of gestures and body movements during virtual learning as sensorimotor activities increased active neural pathways to enhance learning. - The VR environment also offered an opportunity for reflection and repetition to consolidate concepts.	- One of the questions involved understanding how 3D shapes change during a learning task, thought to impose high CL expense. Yet, VR was not beneficial for this particular question, thought to be due to incongruity between the sensorimotor movements employed and the learning content.
- Klippel et al. (2020) - Earth sciences (geoscience)	- Positive impact on learning outcomes. - CL not directly measured.	- Self-reported experiences of learning, enjoyment, and potential benefit were observed from the VR group. - Some benefits were rationalized through pragmatic considerations (e.g., poor weather conditions and hiking) that are circumvented when undertaking a field trip virtually.	- Comparing the two groups, the VR condition described the experience as more educational, compared to the non-VR group, which was seen as more observational. Qualitative responses suggest the VR condition focused attention and guided instruction.
- Lawson and Mayer (2024) - Chemistry (ocean chemistry)	- No impact on learning outcomes. - Positive impact on ECL.	- Executive function was concluded to be related to learning in the VR environment only, with higher executive function correlating with higher achievement. - Individual differences in executive function may be related to learning in VR, but individual differences in WM capacity are not, where learning in VR requires an ability to control one's attention. - Consider that what students feel is distracting them and what actually pulls their attention away likely differs.	- WM capacity is less of a concern than executive function, where learning in VR requires the learner to draw on their executive function, in contrast to the non-VR condition where they did not. - Consider two types of ECL, internal and external, where external (i.e., information outside of the lesson pulling learners' attention away) is more easily identifiable by participants, thus observed in the non-VR group.
- Lyu et al. (2025)	- Positive impact on learning outcomes.	- The use of a spatial reality display improved users' theoretical knowledge and behavioral skills, which was concluded to be more effective in skill transfer from real to virtual scenarios.	- The gesture-based interaction enhanced the engagement and richness of the learning experience.
- Chemistry (chemical reactions)	- CL not directly measured.	- The active participation from the VR condition may deepen memory and understanding, with the novel technology increasing engagement, student interest, and providing increased opportunities for self-directed learning.	- Virtual labs also eliminate safety hazards, potentially unburdening concerns with respect to how students would approach a dangerous experiment in a risk-free environment.
- Martarelli et al. (2025) - Chemistry (water cycle)	- Positive impact on learning outcomes. - Positive impact on total CL.	- Being in the virtual world offered by VR was concluded to be important for fostering the learning process. - The interactive elements (i.e., if the task was taken out by students themselves or via a digital avatar they watched) also investigated had no effect on results, rationalizing that the benefit was canceled out by the constraints of the technology used.	- Whilst CL load was observed to be lower in the VR group, this group was also observed to report a higher physical load (i.e., how much the student used their body), especially when in VR with an avatar. - It was suggested that observing the avatar in VR could have reduced the demands of the task to free up cognitive resources for allocation elsewhere.
- Parmar et al. (2016) - Physics (electric circuits)	- Positive impact on learning outcomes. - CL not directly measured.	- Students in the VR group showed improved outcomes in the “evaluation” level (Bloom's taxonomy) of testing, rationalizing that the viewing condition is important for this level of analysis with psychomotor skills learning. - It is implied that VR may facilitate learning, particularly in a more advanced learning context, so implementation should be carefully considered depending on the level of the cognitive task.	- Differences were observed with the VR group moving the hand-held devices and their heads much more than the non-VR group. Similarly, VR had larger amounts of gaze shift. - This implies students spent more time exploring the virtual environment due to the 360-degree field of regard.
- Sun et al. (2019) - Physics (astronomy)	- Negative impact on learning outcomes. - Positive impact on total CL.	- Performance in the non-VR group was observed to be higher for higher spatial ability learners. - It is rationalized that the use of multisensory channels in VR for these learners, who can already satisfactorily complete the learning task in a non-immersive environment, consumes a large amount of unnecessary cognitive resources.	- Reduction of CL was only observed in the lower spatial ability learners. It is suggested that those with high spatial ability are able to competently generate, maintain, and transform mental representations, whereas students less adept in these abilities require more cognitive resources to generate and maintain external representations. - VR can therefore support this process via multisensory channels to free up cognitive resources that can instead assist in the learning of concepts inherent to the task.
- Trindade et al. (2002) - Chemistry (atomic structure)	- Positive impact on learning outcomes. - CL not directly measured.	- As a learning tool, VR was shown to yield better results when studying phase changes, but there were reservations regarding broader conclusions that could be drawn for the effect of VR stereoscopic visualizations on learning.	- Qualitative analysis confirmed the positive use of VR from the perspective of student opinions that it supported the observation of complex structures.
- Zhao et al. (2022) - Earth science (geoscience)	- Negative impact on learning outcomes. - CL not directly measured.	- The VR group had a worse performance than the non-VR group in learning outcomes from the first field trip; however, this was not observed in the subsequent one.	- It is suggested that the novelty of the highly immersive VR environment imbued a greater level of CL upon first use; yet, this decreased upon subsequent uses due to a decreasing novelty effect.
		- Longitudinal exposure to VR may therefore be beneficial in compensating for the initial deficiency observed in objective learning outcomes. - VR was observed to outperform the non-VR group in other subjective measurements, including enjoyment and satisfaction.	- With less CL imbued in the non-VR group, ample cognitive resources remained to be assigned to observation, comprehension, and recall of knowledge, allowing them to outperform the VR group in the first virtual field trip only.

**Table 6:** Extracted data items for studies that compared two different XR-supported learning conditions.

**Study and discipline**	**Significant effects of XR technology**	**Conclusions on learning outcomes**	**Conclusions on CL**
- Barrett and Hegarty (2016) - Chemistry (molecular geometries)	- Positive impact on learning outcomes. - Positive impact on total CL.	- Students using VR where the object being manipulated was co-located (i.e., in the same position) as opposed to displaced completed trials faster. - Learning with stereo-viewing (that provides 3D depth to the visualization) observed increased response times in VR as well as increased accuracy, specifically when utilized with the co-located VR device. - Students utilizing the VR co-located condition reported lower levels of physical demand, frustration, and rated their own performance higher.	- Higher ability students were observed to be able to compensate for the increased level of CL associated with displaced haptic and visual information. - A threshold may exist where task demands overload cognitive resources of lower spatial ability learners to the point that the high-fidelity interface can no longer compensate for it. - Teaching using interfaces with poor perceptual cues may impose greater load upon lower spatial ability students. It is suggested to use monocular cues when appropriate to avoid additional load demands of stereo-viewing.
- Çeken and Taşkin (2025) - Physics (lightning formation)	- No impact on learning outcomes. - Negative impact on ECL. - No impact on ICL and GCL.	- Differences in learning outcomes between the VR, AR, and non-XR conditions were not observed. - It was concluded that the nature of the subject matter may be congruent with immersive technologies for improved outcomes in this context. - VR was observed to yield higher satisfaction scores, attributed to its immersive and interactive aspects.	- Students utilizing the AR condition experienced more ECL compared to the other technologies, implying a potential novelty effect. - The easy topic of focus, with few learning elements, had such a low ICL as to negate the benefit that the technologies may impose. - The complexity of subject matter and deeper comprehension remained consistent irrespective of setting. A pre-training effect was observed for VR and AR but not for the non-XR control.
- Coban et al. (2025) - Physics (quantum physics)	- Positive impact on learning outcomes. - CL not directly measured.	- The AR condition that received AI feedback increased their learning outcomes compared to those who used AR without AI feedback. - AI utilized not as a source of information but as a tool to guide students to discover information. Engaged learners in effective cognitive learning processes, facilitated by guiding them to the more important elements whilst considering the answer.	- Demonstrated AI can direct and guide students' visual attention via feedback between physical and virtual conditions. - When an experimental procedure contains both physical and virtual components, focusing more on virtual representations while answering conceptual questions may aid in supporting cognition to improve outcomes.
- Huang et al. (2019) - Physics (astronomy)	- Positive impact on learning outcomes. - CL not directly measured.	- Participants in the AR condition showed higher scores on auditory information compared to participants in the VR condition, who showed higher scores on visual information. - Rationalized as VR drawing more attention to visual information in the environment, compared to AR being more effective at conveying information through auditory channels as it frees up cognitive resources that would otherwise be dedicated to visual channels.	- The two modalities placed differing cognitive demands on the user. - With weaker psychological responses in the AR condition to the virtual environment, more cognitive resources were available for retaining auditory information. - In contrast, the VR environment was shown to promote higher levels of attention, spatial presence, and enjoyment.
- Li et al. (2023) - Physics (astronomy)	- Positive impact on learning outcomes. - No impact on ICL, ECL, and GCL.	- Utilizing VR with textual cues yielded higher outcomes compared to using VR without textual cues. Participants using VR with both textual cues and summarizing scaffolds scored higher than all other conditions in measures of mental model and explanation (written, drawing, and detailing). - Using summary scaffolds in VR may require relationships between the information presented and prior learning to be constructed and	- Textual cues utilized in the VR learning environment may provide cues and facilitate learner selection, extraction, and memorization of key information amongst the vast amounts of visual and auditory information that a VR environment can impose. - Suggest no increased level of CL associated with the use of textual cues; however, it may disrupt the level of immersion the user experiences.
		integrated, where deficits in executive functioning may impact the effectiveness of this.	
- Ripsam and Nerdel (2024) - Chemistry (chemical reactions)	- No impact on learning outcomes. - CL not directly measured.	- Observed that utilizing AR on head-mounted glasses tended to positively stimulate chemical terminology, in comparison to using AR on a tablet, which resulted in more behavioral changes concerning understanding of the substance–particle concept. - Overall, using AR evoked greater concentration and a more conscientious approach than using the non-AR version.	- The non-AR and AR conditions differed only slightly in terms of cognitive processing in WM. However, it was suggested that AR on the glasses induced a higher ECL, hindering certain aspects of the learning content.
- Schönborn et al. (2011) - Chemistry (biomolecules)	- Positive impact on learning outcomes. - CL not directly measured.	- Experiencing VR with haptic feedback yielded higher learning gains compared to utilizing VR without haptic feedback. - The force feedback was thought to have offered a sensory opportunity for error correction, where it is the realistic awareness it imparts as a visuohaptic tool that benefits learning more so than only the perception of haptics. - A VR learning experience coordinated with haptics could provide an embodied experience of sub-microscopic processes.	- Haptic feedback allowed offloading from the visual channel to increase the cognitive capacity available to link new mental representations to pre-existing knowledge. Correspondences between the visual and tactile modes would alleviate demand on the visual pathway alone, freeing up WM resources for learning. - Learners with low spatial ability required high amounts of cognitive resources toward manipulating multiple mental visual representations, where haptic feedback could compensate for this.
- Seo and So (2022) - Physics (electric circuits)	- No impact on learning outcomes. - CL not directly measured.	- No difference observed between the AR condition that offered guidance and the AR condition that did not. This was rationalized by the careful consideration between concepts and the gesture-based AR exhibit used.	- If the AR learning environment is already carefully designed through consideration of concepts and gestures, then additional devices such as guidance may not be necessary to reduce CL, as it already allows easy recognition and interaction.
- Vogt et al. (2021) - Chemistry (ocean chemistry)	- No impact on learning outcomes. - CL not directly measured.	- Learning using VR with or without annotations did not reveal any difference in learning. - The helpfulness of annotations, and the level of learning outcome, they benefit, depends on the information displayed and the composition of the different representations of learning material, where annotations with relational info may be helpful to integrate a coherent mental model.	- It was suggested that learners with high intrinsic motivation, who outperformed those with low-intrinsic motivation, invested a higher level of cognitive resources through more efficient search patterns that compensated for the lack of annotations. - Annotations, therefore, could guide and support attention, reducing visual search, and freeing up cognitive resources for less motivated learners.
- Wang et al. (2024a) - Physics (electric circuits)	- Positive impact on learning outcomes. - Positive impact on ECL and GCL. - No impact on ICL.	- Utilizing VR with feedback yielded higher learning outcomes in measurements of knowledge, comprehension, and application compared to using VR without feedback. - Students who did not receive feedback were thought to construct knowledge out of sequence, impeding the higher-order cognitive processing that occurs for those who did.	- No change in ICL expected as feedback did not change the inherent task difficulty. ECL and GCL were both improved in feedback groups. - Feedback negated the potential for participants to become stuck in trial-and-error loops. When it was generated after the learner interacted with the environment, it was thought to reduce ECL by diminishing the need for unnecessary visual searching.
- Wang et al. (2024b) - Physics (electric circuits)	- Positive impact on learning outcomes.	- Students who utilized VR with feedback were observed to obtain higher levels of cognition, learning motivation, and self-regulated learning	- Real-time feedback in VR may have the ability to improve cognitive capacity in applying acquired knowledge to real-world scenarios. Additionally, it
	- CL not directly measured.	abilities to yield greater improvements compared to those using VR without feedback. - Feedback allowed for immediacy in understanding that allowed students to make appropriate adjustments and track their learning.	can guide the user to reduce visual load through unnecessary searching.
- Wiebe et al. (2009) - Physics (forces)	- Positive impact on learning outcomes. - CL not directly measured.	- Observed that utilizing VR without haptic feedback yielded higher results on an embedded learning assessment compared to using VR with haptic feedback, which also increased the total fixation time on key elements. - Rationalized that the visual information contained in the numeric readout was preferable to using haptic feedback.	- A high CL cost was already imposed from a number of factors, including the inherently complex learning task, high element interactivity of the novel interface, and perceptual demands of the digital exploration task. - This rescinded the ability to appropriately integrate the visual and haptic information to benefit the learning process.
- Xie et al. (2023) - Physics (electric circuits)	- Positive impact on learning outcomes. - No impact on ECL and GCL.	- Both the VR condition with corrective feedback and the VR condition with explanatory feedback improved learning outcomes compared to the non-VR condition. No difference was observed between the two VR conditions themselves. - Concluded that VR had a positive effect on intrinsic motivation, leading to higher academic achievement. Yet the lack of difference between the two VR conditions was due to visual cues, as opposed to textual cues, being more influential in a VR environment.	- Inferred that the amount of information provided by the VR environment should not affect cognitive processing, provided that the feedback is closely tied to the learning activity. - A negative correlation was observed between ECL and both GCL and learner satisfaction, implying that those under higher ECL will be less likely to engage in active schema construction, reducing their satisfaction with the VR environment.

**Figure 6:**
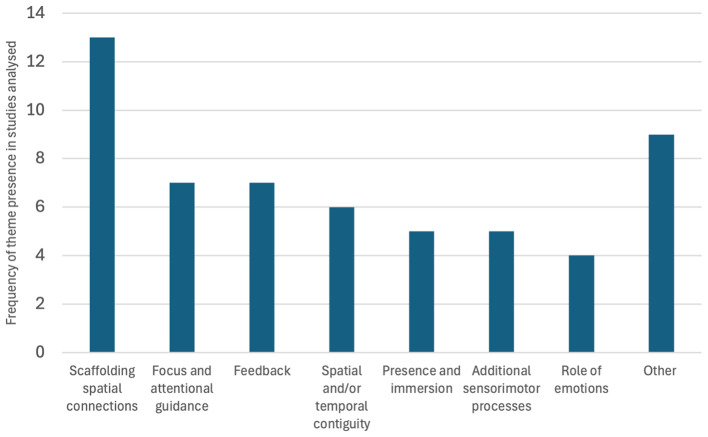
Frequency of the presence of themes identified in the studies analyzed (themes with a frequency of 3 or less have been combined as “other”).

The studies were then analyzed to ascertain the implications drawn on visuospatial processing with respect to the comparative XR learning intervention that was implemented (refer to Table 7). These were subsequently coded, with each theme available to be assigned a maximum of once per study, in order to ascertain the prevalence of the different themes regarding XR impact on visuospatial abilities throughout this body of research (refer to Figure 6).

**Table 7:** Extracted data items for all studies considering visuospatial processing based on the comparative XR intervention.

**Study**	**Comparative XR learning conditions**	**Implications on visuospatial processing**	**Theme(s) identified**
Altmeyer et al. (2020)	- AR-supported vs. - Non-AR	- The spatially de-located but real-time measuring display AR condition resulted in higher conceptual knowledge.	- Spatial and/or temporal contiguity
Amirbekova et al. (2024)	- VR-supported vs. - Non-VR	- Memory and visuospatial skills were observed to improve in the VR group.	- Improving visuospatial skills
Arymbekov et al. (2024)	- AR-supported (with pre-test) vs. - AR-supported (no pre-test) vs. - Non-AR (with pre-test) vs. - Non-AR (no pre-test)	- AR could facilitate establishing spatial connections aligning with the cognitive developmental stage of the student. - May enhance 3D skills, especially for weaker visual perception students.	- Scaffolding spatial connections - Improving visuospatial skills
Barrett and Hegarty (2016)	- VR-supported (co-located) vs. - VR-supported (displaced)	- High spatial ability learners with the co-located VR device had faster response times. - Beneficial effects of providing stereo-viewing (as opposed to monoscopic) likely only arise when binocular depth cues afford an additional task relevance. - State the importance of co-located interaction in VR for more difficult rotation tasks, especially for low to average spatial ability students.	- Spatial and/or temporal contiguity - Depth of digital environment
Çeken and Taşkin (2025)	- VR-supported vs. - AR-supported vs. - Non-XR	- Attribute the increase in ECL shown in the AR condition in part to the challenge of focusing amidst the overlay of digital information onto the natural world. - Modality effect observed between students using visual and auditory modes in AR but not VR, attributed to multiple factors such as the specific learning environment and subject.	- Focus and attentional guidance
Coban et al. (2025)	- AR-supported (AI feedback) vs. - AR-supported (no AI feedback)	- Eye gaze tracking data revealed that receiving audio feedback led to visual attention being favored more toward the real experimental equipment for process-oriented activities, compared to attention being favored toward the virtual elements in concept-related questions.	- Feedback
Crogman et al. (2025)	- VR-supported vs. - Non-VR	- The immersive nature of VR allowed students to visualize and manipulate physical systems to facilitate a deeper understanding of the underlying principles.	- Presence and immersion
Elford et al. (2023)	- AR-supported vs. - Non-AR	- Qualitative interviews suggest participants identified the presence of beneficial visual design of the technology, e.g., in the positioning of learning elements close to one another.	- Spatial and/or temporal contiguity
Elford et al. (2022)	- AR-supported vs. - Non-AR	- Qualitative analysis revealed students perceived that AR assisted the visualization process. - Yet they perceived they needed to invest similar levels of cognitive effort to comprehend the molecular geometry representations irrespective of group.	- Scaffolding spatial connections
Ernawati and Ikhsan (2021)	- VR-supported vs. - Non-VR	- Students were able to more actively develop their imaginative ability when using the virtual environment compared to those who undertook the conventional lab.	- Scaffolding spatial connections
Habig (2020)	- AR-supported vs. - Non-AR	- Mental rotation scores were observed as a covariate on test scores. This was also observed to increase the effect of sex as a variable on outcomes.	- Gender
Huang et al. (2019)	- VR-supported vs. - AR-supported	- Students' feelings of spatial presence played a mediating role between modality and learning. In the VR environment, more visual attention was paid to the mediating environment; thus, users received more information of the same modality, i.e., visual. - Similarly, scores on auditory-related information were mediated by spatial presence, where higher levels of spatial presence in VR accounted for the lower auditory scores achieved and the ultimate outperformance by the AR group in this metric.	- Presence and immersion - Focus and attentional guidance - Additional sensorimotor processes
Keles et al. (2023)	- VR-supported vs. - Non-VR	- Sensorimotor activities were high during the virtual experience, suggesting its benefit in adding to the visual and auditory modalities in the learning of concepts that involve 3D information in a VR environment.	- Additional sensorimotor processes
Keller et al. (2021)	- AR-supported vs. - Non-AR	- A relationship was observed between mental rotation abilities and post-test scores in the non-AR group but not in the AR group on certain learning tasks. - This may imply the ability for AR to reduce the influence of mental rotation abilities on learning gains.	- Scaffolding spatial connections
Kenneally and Bentley (2024)	- AR-supported vs. - Non-AR	- AR allowed students to apply mental rotation visualizations with less obstruction, whereas the non-AR group utilized more WM resources in this process, which impeded their ability to create and transfer new schemas. - Mental rotation has an effect on CL, but CL can be altered using AR technology.	- Scaffolding spatial connections
Klippel et al. (2020)	- VR-supported vs. - Non-VR	- The elevated perspective offered by the VR experience provided the ability to explore from a perspective not usually feasible, with analysis of spatial situation models showing differences favoring this condition.	- Offering new perspectives
Laumann et al. (2024)	- AR-supported vs. - Non-AR	- Considered the extent to which lab courses (such as the focus of this study) are suitable for the development of content knowledge utilizing AR. It was assumed that spatial abilities were not particularly important for this topic and thus not appropriate for AR use. - The additional AR visualization may have been perceived as increasing the complexity of the learning environment, thus requiring more WM resources to make sense of the representations within the context of the learning task.	- Appropriateness of learning content
Lawson and Mayer (2024)	- VR-supported vs. - Non-VR	- A learner with a weaker executive function is more likely to be impacted by lessons with higher potentials for distraction, where the cognitive process of selecting may be particularly challenging in visually rich environments such as VR.	- Executive function role
Ling et al. (2021)	- AR-supported vs. - Non-AR	- Proposed that students with high spatial abilities and a solid learning foundation can benefit from AR to improve lasting learning outcomes, irrespective of their attitude toward the technology. - Without high spatial abilities, it is recommended that the learner is passionate about the technology.	- Role of emotions
Liou et al. (2017)	- AR-supported vs. - Non-AR	- Perceived usefulness was higher in the AR group as they could use the objects in the real environment as a reference to describe the position of the moon in terms of direction and elevation. - The non-AR group were unable to do this, instead needing to imagine the virtual scenario in the real environment, making it harder to remember the movement of the moon.	- Scaffolding spatial connections
Liu et al. (2021)	- AR-supported vs. - Non-AR (digital lab) vs. - Non-AR (traditional lab)	- The full characteristics of AR were utilized, particularly with respect to the spatial and temporal contiguity of information, allowing a reduction in students' perceived task difficulty. - Integration of AR reduced the cognitive demands on students allocated to the learning task by providing a virtual-real fused environment to assist the construction of knowledge.	- Spatial and/or temporal contiguity - Scaffolding spatial connections
Li et al. (2023)	- VR-supported (textual cues/summarizing scaffolds) vs. - VR-supported (no textual cues/summarizing scaffolds)	- Using verbal summaries in a VR learning environment may be effective for text-based knowledge, but pictorial summaries are potentially more appropriate for knowledge concerning complex spatial representational relationships.	- Feedback
Lyu et al. (2025)	- VR-supported vs. - Non-VR	- The spatial reality display utilized provided a more realistic experimental experience, effectively enhancing memory and understanding.	- Improving visuospatial skills
Martarelli et al. (2025)	- VR-supported (high immersion) vs. - VR-supported (low immersion) vs. - Non-VR (high immersion) vs. - Non-VR (low immersion)	- Spatial presence was observed to be higher in the VR condition, suggesting the feeling of “being there” was an important source of learning. - Higher physical load was reported when the user was immersed in the virtual environment, inferring that VR promoted a greater use of the body during learning.	- Presence and immersion - Additional sensorimotor processes
Olea-Ibarra et al. (2025)	- AR-supported vs. - Non-AR	- Immersive media may be particularly detrimental if they evoke emotions distracting from the task, possibly as it was cognitively overwhelming.	- Role of emotions
Parmar et al. (2016)	- VR-supported vs. - Non-VR	- The disconnect of the computer screen in the non-VR condition could impose a visual disconnect that is jarring to students, consequently affecting their closed-loop visual-motor response to adjust to the environment. The VR group was observed to adjust much quicker. - Distortion of artifacts in the non-VR condition at certain angles (that did not occur in the VR group) could also have been an influencing factor in users' favoring of the VR technology.	- Spatial and/or temporal contiguity
Ripsam and Nerdel (2024)	- AR-supported (glasses) vs. - AR-supported (tablet) vs. - Non-AR	- The use of the AR conditions may have initiated cognitive processing based on multiple external representations and symbols, favoring the development of both a mental and a text-based image. - AR has the potential to avoid split attention and link the material level spatially and temporally with the virtual processes investigated.	- Spatial and/or temporal contiguity
Schönborn et al. (2011)	- VR-supported (haptics) vs. - VR-supported (no haptics)	- Students with higher spatial abilities engaged the different visual modes on offer less, suggesting that haptic feedback may offer a visuo-tactile map for physically realistic movement. - In contrast, the no-haptic group switched frequently, implying their need to rely more heavily on viewing the features, necessitating the need to hold multiple mental visualizations in visual WM simultaneously.	- Feedback - Additional sensorimotor processes
Seo and So (2022)	- AR-supported (guided) vs. - AR-supported (non-guided)	- Visual guidance did not enhance conceptual understanding; rather it was meaning-making through verbal interaction in pairs that was the more significant factor influencing the learning process.	- Focus and attentional guidance
Singh and Kaur (2025)	- AR-supported vs. - Non-AR	- The use of the AR app allowed exploration of spatial relationships inherent to the 3D nature of the content covered.	- Scaffolding spatial connections
Sotiriou and Bogner (2008)	- AR-supported vs. - Non-AR	- AR visualizations allowed students to relate their actions to representations that supported and scaffolded thinking. - This visualizing of the phenomena was thought to provide links between the student's real-life exploration and the abstract representations that the exhibit presented.	- Scaffolding spatial connections
Sun et al. (2019)	- VR-supported vs. - Non-VR	- Low spatial ability learners consume higher levels of CL in generating and maintaining external representations, where they were unable to do this adequately in the non-VR condition with static visual information. Yet the dynamic VR condition complemented the process of spatial information transformation, reducing additional levels of CL that can instead be utilized on the embellishment of mental models. - Conversely, neither condition required higher spatial ability learners to exceed their total cognitive resources, so that compensation from VR was deemed to be unnecessary in this context.	- Scaffolding spatial connections
Thees et al. (2022)	- AR-supported vs. - Non-AR	- The type of display between the two groups may have affected how participants solved the problems. The symbolic circuits may have been less relevant to the non-AR group as they assigned less of their visual attention to this during the	- Focus and attentional guidance
		experiments. Instead, they focused more on the displayed measures on the tablet, performing simpler local coherence formation (which the AR group failed to do). - If local coherence formation could be achieved in the AR group, facilitation into global coherence may have been able to occur.	
Thees et al. (2020)	- AR-supported vs. - Non-AR	- Multiple external representations offered complementary approaches, provided different levels of accuracy, and addressed different aspects of the scientific phenomena. - AR also provided chances to compare the quantitative visualization and qualitative observation of the same target.	- Scaffolding spatial connections
Trindade et al. (2002)	- VR-supported vs. - Non-VR	- Correlations were observed between conceptual comprehension of subjects and most visualization process parameters. Qualitative analysis suggested the VR condition supported the understanding of 3D behaviors.	- Scaffolding spatial connections
Utami et al. (2020)	- AR-supported vs. - Non-AR	- Qualitative analysis revealed that students liked using the AR media that displayed 3D visuals representing the phenomena being studied.	- Role of emotions
Vogt et al. (2021)	- VR-supported (annotations) vs. - VR-supported (no annotations)	- Suggest that to reveal a substantial benefit from annotations in a VR environment, the material needs to be sufficiently complex, as opposed to the simple depictions of this study, where lowering the visual requirements may negate the beneficial effects as signaling would no longer be required.	- Appropriateness of learning content - Focus and attentional guidance
Wan et al. (2018)	- AR-supported vs. - Non-AR	- The visual provisions of the AR components, i.e., videos, animations, images, helped students to acquire a better understanding of the lessons as they could see phenomena that they normally could not in the lab.	- Scaffolding spatial connections
Wang et al. (2024a)	- VR-supported (with feedback) vs. - VR-supported (without feedback)	- Participants thought to be distracted when trying to visually find components without feedback, which was able to maintain participant engagement. - Achieved this by guiding the user to lock on to the appropriate target to reduce the visual search load.	- Feedback - Focus and attentional guidance
Wang et al. (2024b)	- VR-supported (with feedback) vs. - VR-supported (without feedback)	- Gaze feedback assisted participants in reducing retrieval time in VR to mitigate visual load, ultimately contributing to higher self-regulated learning and motivation.	- Feedback - Focus and attentional guidance
Wiebe et al. (2009)	- VR-supported (haptics) vs. - VR-supported (no haptics)	- Using haptic feedback significantly increased the amount of time spent fixating on elements in the VR environment relative to the other portions of the digital interface. - Suggested that when vision is available and adequate for a task, haptic exploration may not be evoked due to its high processing cost.	- Feedback - Additional sensorimotor processes
(Xie et al. 2023)	- VR-supported (corrective feedback) vs. - VR-supported (explanatory feedback) vs. - Non-VR	- Increased learning motivation thought to be related to the immersive 3D features of VR. Yet the stronger sense of immersion and presence necessitates a balance to be obtained between high-immersion requirements and the specific educational goals it is aiming to achieve. - Suggest that the text-based features between the two VR conditions could not engage students in deep learning as the environment inherently engages with images, not text, due to its visual nature.	- Presence and immersion - Feedback
(Zatarain-Cabada et al. 2023)	- AR-supported vs. - Non-AR	- Presentation of 3D audiovisual elements in the AR condition seemed to initially overwhelm students. - Yet assert that the technology has the potential to offer a more interactive and intuitive learning experience.	- Role of emotions
(Zhao et al. 2022)	- VR-supported vs. - Non-VR	- Students reported significantly higher levels of presence in the VR condition. - Yet the perceptual realism and richness of sensory information in the	- Presence and immersion
		immersive VR condition, whilst enhancing emotional engagement, may impose high CL costs that could distract from the processing of essential materials.	

## 4 Discussion

### 4.1 Learning outcomes and cognitive load

Upon analysis of the learning outcome data extracted from each study, benefits in the use of XR technology were found (refer to Figure 5). Sixty-three percent (*n* = 27) of studies analyzed identified that the relationship between XR use and improvement to learning was statistically significant. This is in contrast to only 9% (*n* = 4) of studies concluding a statistically significant negative impact of XR technology on learning and 28% (*n* = 12) of studies that found no difference. When considering studies that only reported a measurement of total CL, 86% found that XR technology had achieved a statistically significant positive impact vs. no reports of significant negative impacts. Similarly, when considering the studies that reported on individual CL types (i.e., ICL, ECL, and GCL instead of total CL), it was observed that additional studies reported statistically significant positive impacts (*n* = 6) with only a minimal number of these with negative impacts reported (*n* = 2). Yet with respect to individual measurements of CL (i.e., ICL, ECL, and/or GCL), most studies concluded a non-significant effect of the technology use on the individual measurements of CL (*n* = 25). While the reason for this remains inconclusive, and may require further investigation, the findings answer RQ1 to affirm that XR technologies can reduce CL and benefit learning outcomes when applied to a learning context requiring visuospatial abilities in the physical sciences.

### 4.2 Level of presence and successful XR implementation

The studies that reported significant impacts on learning outcomes and/or CL when implementing an AR-supported intervention (*n* = 15 positive, *n* = 4 negative) or VR-supported learning intervention (*n* = *16* positive, *n* = 2 negative) were analyzed to ascertain how, if at all, the extent to which XR elicits student experience of spatial presence and XR technology offers impacts successful implementation.

Concerning effective AR use, multiple external representations in the AR environment did not inherently increase the complexity of the material, yielding no impact on ICL, instead effectively decreasing ECL (Thees et al., 2020). This benefit was observed to occur in a number of ways: supporting the visualization process to reduce the impact of pre-existing spatial abilities (Keller et al., 2021; Kenneally and Bentley, 2024), allowing immediacy and connection with realistic contexts (Liou et al., 2017), utilizing visualizations to correct misconceptions (Sotiriou and Bogner, 2008), or by transforming abstract concepts into concrete ones (Liu et al., 2021). However, it was noted that there is a need to ensure the inherent complexity of the content does not change, i.e., that the ICL remains constant with the addition of the technology aiming to facilitate ECL reduction. Ineffective AR design was observed to increase ICL when the visualizations imposed into the digital environment increased the level of complexity (Laumann et al., 2024), thus increasing the number of elements that the learner had to consider simultaneously. The particular AR medium itself (e.g., tablet, glasses, mobile device) did not seem to be a factor in successful implementation (Altmeyer et al., 2020), but ensuring that there was congruency between the technology used and the cognitive processes it is aiming to trigger did (Çeken and Taşkin, 2025; Thees et al., 2020), with poor tech design correlated with an increased level of ECL to the detriment of the task design (Keller et al., 2021). This is particularly important in terms of ensuring usability in an organized and intuitive way to facilitate curiosity and motivation for learning (Zatarain-Cabada et al., 2023) and through the incorporation of different learning types to leverage the learning modalities available (Wan et al., 2018).

Beyond CL effects, the literature postulated that VR implementation improved student learning outcomes due to the depth of learning it offers through enhanced engagement (Klippel et al., 2020; Lyu et al., 2025; Xie et al., 2023). Even Zhao et al. (2022), who reported a negative impact on learning outcomes compared to its non-VR comparator, still observed it outperforming in user metrics of enjoyment and satisfaction. Facilitated by these intensified engagement levels, VR appears to yield its benefit through its multifaceted ways of providing realistic simulations, rich visualization, and abstract conceptualization to construct and apply mental models (Crogman et al., 2025; Ernawati and Ikhsan, 2021), yet the effect on learning outcomes may still be somewhat debated. It was also suggested that VR's full potential emerges in relatively more advanced learning contexts (Parmar et al., 2016) with careful consideration between VR implementation and the corresponding appropriateness of task complexity a requirement for successful implementation (Sun et al., 2019; Trindade et al., 2002). Recent research by Lawson and Mayer (2024) asserted that individual differences in learner executive function, as opposed to inferences for WM capacity itself, are related to learning in a VR environment, where learning in VR requires users to utilize executive function in order to control their attention toward the salient learning elements in the digital environment, a factor less important in a non-VR alternative. Li et al. (2023) echoed this proposed relationship with respect to deficits in executive functioning and the impact that this can have on learning in a VR environment, a concept that would benefit from further investigation.

### 4.3 XR to alleviate visuospatial CL

RQ2 concerned the impact of XR technology on visuospatial processing. Figure 6 shows that the most frequently identified visuospatial processing theme was “scaffolding spatial connections” (*n* = 13). Overall, 77% of these studies were observed to implement an experimental design between an AR-supported condition and a non-AR comparator. Of these, all but one observed at least one significant positive impact of the use of AR on measurements of learning outcomes and/or CL, strongly implying a mechanism through which this type of XR technology supports cognition. Liu et al. (2021) observed positive metrics in both of these outcomes, rationalizing that fusing the virtual and real environments assists in alleviating the visual cognitive burden imposed by the learning task, freeing up cognitive resources otherwise unavailable to assist in the construction of knowledge. Conclusions were also drawn on the potential for AR to reduce the influence of mental rotations on learning gains (Keller et al., 2021; Kenneally and Bentley, 2024) and facilitate referential connections between the learner's real-world experience and the abstract virtual learning content (Liou et al., 2017; Sotiriou and Bogner, 2008; Wan et al., 2018).

“Spatial and/or temporal contiguity” was the other theme identified to disproportionately favor importance in AR (*n* = 6), with 67% of these particular studies implementing an AR intervention. Successful implementation here was considered through AR's ability to avoid split attention, linking the digital material in both space and time with the real environment it is imposed upon (Ripsam and Nerdel, 2024). With elements of the real world still present within an AR interface, the design of the learning task should therefore aim to spatially and temporally locate the learning elements close to one another to reduce the cognitive resources otherwise spent unnecessarily during the learning task (Altmeyer et al., 2020; Elford et al., 2023; Liu et al., 2021).

The themes of “focus and attentional guidance” and “feedback” were the second most commonly identified (*n* = 7 for both). Interestingly, of these 14 occurrences, 13 of them were observed in interventional studies comparing two different types of XR-supported learning environments. Visual cueing was the main form of attentional guidance discussed. In VR, visually signaling the salient learning outcomes was observed to reduce the visual searching required to be performed by the learner, ultimately reducing visual load to promote a higher level of self-regulated learning and motivation (Wang et al., 2024a,b). This is in contrast to AR, with Thees et al. (2022) observing that visual attentional guidance in AR resulted in a significant negative impact on learning outcomes. Similarly, Çeken and Taşkin (2025) observed that participants in a visually guided AR condition experienced significantly higher levels of ECL compared to both VR and non-XR comparators. These findings become particularly interesting within the context of Huang et al. (2019), who investigated learning outcome performance between AR and VR using two separate parameters: auditory knowledge and visual knowledge. Participants in the AR condition were observed to score significantly higher on the auditory information retention assessment compared to VR participants who scored significantly higher on the visual assessment. These authors assert that the two environments place different cognitive demands on the user. In VR, more visual attention is required for the mediating environment, causing the learner to receive more information in this same modality (i.e., visual). In contrast, with weaker responses to the lower amount of visual information imposed by an AR environment, more cognitive resources remain available to retain auditory information (Huang et al., 2019). Similar trends can be observed in terms of the effect of “feedback” in virtual environments. Visual text-based feedback was observed to yield significant positive outcomes when applied in VR (Li et al., 2023; Xie et al., 2023). Coban et al. (2025) observed a significant positive effect on learning outcomes where an AR-supported condition that received feedback outperformed another AR-supported condition that did not. In this study, participants could verbally direct questions to an AI model that utilized its text-to-speech function to, crucially, provide auditory feedback that guided the task. Overall, these results suggest that the use of feedback and attentional guidance may be most effective in a VR-supported learning context when given in a visual form, compared to an AR-supported condition that benefits from an audio form.

The themes of “presence and immersion” (*n* = 5) and “additional sensorimotor processes” (*n* = 5) were observed to occur exclusively in studies that utilized a VR learning intervention. Perhaps unsurprisingly, being immersed within the virtual environment was identified as particularly important for learning in VR, where the importance of “being there” resonated throughout the studies of this nature. Achieving this level of presence has the potential to evoke a deeper understanding, facilitate visualization and manipulation of concepts, and increase learning motivations (Crogman et al., 2025; Martarelli et al., 2025; Xie et al., 2023). Yet care needs to be taken to avoid high CL costs that can be imposed by such sensory-rich environments, e.g., Zhao et al. (2022), who observed a significant negative impact on learning outcomes in a VR-supported condition compared to a non-VR comparator upon the first time participants used the technology. Yet upon considering longitudinal effects, these authors observed that this negative effect was not present in subsequent VR use, suggesting that novelty effects could exist upon initial VR use that are subsequently mitigated with repeated exposure to allow VR's high level of presence to more actively impose beneficial effects. Finally, the impact that sensory input, beyond only visual and spatial, could have upon additional sensorimotor processes when learning in a VR environment was an interesting find. Beyond the implication of auditory information already discussed, the use of gestures and body movement as sources of additional sensory input available when utilizing VR as a head-mounted display to simulate neural pathways to benefit learning was considered (Keles et al., 2023; Martarelli et al., 2025). As was the use of human movement via haptic sensory input as a way to increase cognitive capacity in a learning task. Schönborn et al. (2011) demonstrated that the use of VR with haptic input could improve learning outcomes when compared to a VR comparator that had these haptic functions disabled. Participants in the non-haptic environment were observed to switch between the different visual modes offered by the VR interface more frequently compared to those who had haptics enabled, implying that they were more reliant on visuospatial processing and required to hold multiple mental visualizations in visual WM simultaneously. The authors concluded that the body movements obtained from the haptic feedback allowed offloading from the visual channel to occur, freeing up cognitive resources that could instead be used to link pre-existing cognitive models with new mental representations in schema construction. Yet broader conclusions regarding haptic feedback may have to remain inconclusive. Wiebe et al. (2009) observed the inverse, with their non-haptic VR condition outperforming its VR haptic counterpart; however, the authors did conclude that the high CL cost already imposed by the complex task may have rescinded the ability of the learner to appropriately integrate the visual and haptic information to benefit learning. Strengthening the conclusions regarding body movement in XR environments would be another interesting avenue for future research.

### 4.4 Theoretical implications and limitations

Considering the implications of the studies analyzed, the characteristics and properties of successful implementation of XR-supported learning in the physical sciences can be suggested. In terms of AR-supported learning, benefits appear to be yielded due to the scaffolding of the spatial connections inherent in the visuospatial information presented in a manner that is spatially and temporally contiguous, allowing cognitive resources to be freed up for use in schema construction. AR implementation can also benefit from effective implementation of auditory attentional guidance and feedback. In contrast, successful implementation of VR-supported learning appears to be achieved through the sense of presence that it can impart upon a user, where care needs to be taken to avoid potential visual overload, especially for novices to the technology. VR implementation can benefit most from effective implementation of visual attentional guidance and feedback.

This systematic literature review only incorporated research from academic journals, potentially limiting the conclusions drawn, where a broader consideration from other sources, such as conferences or book chapters, may also yield valuable insight that could further strengthen conclusions, especially at a time of rapid increase in XR studies in the physical sciences (see Figure 3). Similarly, the restrictions on the methodological requirements, e.g., through necessitating a comparative analysis as the intervention for study inclusion, may have also limited the conclusions that could be drawn had studies with additional methodological designs also been included. While this current study provided further information to the field of study, a number of questions remain unresolved and gaps unaddressed. Future research is required to investigate the effect that the use of body movements and gesturing has in relation to offloading CL, as well as in better understanding the potential role of executive function in optimizing learning in these types of virtual environments, especially with respect to VR. Comparing and contrasting the benefits of XR between specific types of learning outcomes more explicitly (e.g., conceptual understanding, skills-based, and transfer tasks) would also be an avenue of further research that would offer valuable insight into this emerging field of research.

## 5 Conclusion

This systematic literature review provides an understanding of the relationship between visuospatial skills, the amount of CL a learner experiences, and the type of XR technology utilized in order to optimize learning when applied to a physical science learning context. The study investigated and analyzed *n* = 44 studies. The systematic literature review found that XR technology can be applied to physical science learning contexts that require the use of visuospatial abilities to reduce CL and improve learning outcomes. The benefit of AR as a form of XR prompting a lower level of experienced presence is suggested to manifest via the imposition of digital elements upon a real environment. The benefits of AR are attributed by the literature to a high degree of spatial and temporal contiguity in a manner that will not impose any additional ICL, offering a mechanism that can scaffold the salient spatial connections inherent in the visuospatial information of the learning task. The more benefit of greater presence offered by VR manifests through its ability to saturate a user into an entirely new and different virtual environment, generating high levels of enthusiasm that facilitate a depth of learning that may be more conducive to learning relatively more advanced concepts. With such a visually stimulating environment, visual attentional cues and feedback appear to be more effective in VR, where, upon a spectrum of XR technology, as the technology offers less presence to instead embody AR, auditory attentional cues and feedback instead become more beneficial. Future research needs to continue strengthening the conclusions regarding best practice in this area and the CL effects that are available to be taken advantage of when using XR technologies. Through careful consideration of the type of XR technology being implemented and its relationship to the goals of the learning task, the results and conclusions drawn in this systematic review can help inform best practice with respect to optimizing XR task design in terms of CL when applied to physical science learning requiring demands on visuospatial abilities.

## Data Availability

The original contributions presented in the study are included in the article/supplementary material, further inquiries can be directed to the corresponding author.
